# Insulin resistance as a predictor of long-term adverse cardiovascular event risk in patients with atrial fibrillation following radiofrequency catheter ablation

**DOI:** 10.3389/fendo.2026.1831673

**Published:** 2026-07-02

**Authors:** Hanxiong Liu, Junli Pan, Yan Luo, Yan Tang, Dongyue Jia, Jie Feng, Yuqi Tao, Sisi Wang, Shiqiang Xiong, Wei Huang

**Affiliations:** 1 Department of Cardiology, The First Affiliated Hospital of Chongqing Medical University, Chongqing, China; 2 Department of Cardiology, The Third People's Hospital of Chengdu, (Affiliated Hospital of Southwest Jiaotong University), College of Medicine, Southwest Jiaotong University, Chengdu, Sichuan, China; 3 Department of Cardiology, The Third People's Hospital of Chengdu, Affiliated Hospital of Southwest Jiaotong University, Chengdu Cardiovascular Disease Research Institute, Chengdu, Sichuan, China

**Keywords:** atrial fibrillation, catheter ablation, insulin resistance, major adverse cardiovascular events, risk prediction

## Abstract

**Background:**

Catheter ablation is the first-line therapy for atrial fibrillation (AF), yet a significant proportion of patients experience major adverse cardiovascular events (MACEs) post-ablation, highlighting a need for improved risk stratification.

**Objective:**

This study aimed to evaluate the association between four non-insulin-based insulin resistance (IR) indices and long-term MACEs in AF patients undergoing radiofrequency catheter ablation (RFCA), and to determine their incremental predictive value beyond established clinical risk factors.

**Methods:**

A retrospective observational study was conducted on 922 non-valvular AF patients who underwent index RFCA between March 2017 and July 2023. The primary endpoint was a composite of MACEs, defined as all-cause death, late AF recurrence, heart failure events, and stroke occurring after the 3-month blanking period. Associations between the four IR indices and MACEs were examined using multivariable Cox proportional hazards regression, restricted cubic spline analysis, and Kaplan-Meier survival estimation. The incremental predictive value of each index over a baseline clinical model was quantified using the C-statistic, net reclassification improvement (NRI), and integrated discrimination improvement (IDI).

**Results:**

Over a median follow-up of 36 months, MACEs occurred in 242 patients (26.25%). After full multivariable adjustment, METS-IR and TyG-BMI were independently and significantly associated with elevated MACE risk across all models (both P < 0.001), while the TG/HDL-C ratio and TyG index did not reach statistical significance in fully adjusted models. METS-IR demonstrated an approximately linear dose-response relationship with MACEs on restricted cubic spline analysis. When added to the baseline clinical model (C-statistic: 0.702), METS-IR conferred the greatest incremental predictive gain, significantly improving the C-statistic to 0.717 (P < 0.001), alongside meaningful NRI (0.291, 95% CI: 0.126–0.426, P < 0.001) and IDI (0.015, 95% CI: 0.004–0.035, P = 0.004).

**Conclusion:**

Among the evaluated non-insulin-based IR indices, METS-IR demonstrated a statistically significant, albeit modest, incremental improvement in the prediction of composite MACE in AF patients following RFCA. These findings support the use of METS-IR in post-ablation risk assessment and may contribute to improved patient outcomes.

## Introduction

Atrial fibrillation (AF), the most common sustained cardiac arrhythmia, affects approximately 1% to 2% of the overall population (1). Since AF increases the risk of stroke, heart failure (HF), and mortality, the disease is a significant contributor to cardiovascular hospitalization and a major threat to public health (2). Catheter ablation (CA) has been demonstrated as an evidence-based, safe, and effective treatment for reducing AF burden, improving quality of life, delaying the progression from paroxysmal AF to persistent AF, and enhancing the prognosis of AF patients with HF (3–6). Consequently, CA has been recommended as the first-line therapy for AF (7, 8). Unfortunately, a significant proportion of patients still experience late AF recurrence and unfavorable outcomes following CA. Thus, the development of prediction models for stratifying the risk of adverse outcomes in AF patients undergoing CA remains a clinical unmet need.

Accumulating evidence indicates that metabolic disorders can promote direct changes to the atrial myocardium, forming the substrate for the development of AF through structural and electrophysiological remodeling (9). Among these metabolic disturbances, insulin resistance (IR), a pathological condition characterized by impaired or diminished responses to insulin in cells or tissues, is a typical characteristic of metabolic disturbances, such as overweight or obesity, diabetes mellitus (DM), and metabolic syndrome. A growing body of studies has demonstrated a correlation between IR and the new onset of AF, as well as an elevated risk of AF recurrence following CA (10, 11). The relationship between IR and long-term adverse outcomes in AF patients undergoing CA remains unestablished.

The objective of the present study was to investigate the association between IR indices and the long-term adverse outcomes, encompassing all-cause mortality, late AF recurrence, HF events, and stroke following RFCA in patients with AF.

## Methods

### Study design and participants

This study shares the same institutional registry with previously published work from our group [Luo et al., 2024 (12); Qin et al., 2025 (13)]. The present analysis differs substantively in its primary endpoint (composite MACE including hard cardiovascular events), study period (extended to July 2023), and analytic focus on the incremental predictive value of IR indices beyond established clinical risk factors.

A retrospective observational study was conducted at the Third People’s Hospital of Chengdu (Sichuan, China) between March 2017 and July 2023. The study included patients with non-valvular AF admitted for index RFCA treatment. Exclusion criteria comprised: (1) prior left intra-atrial CA and left atrial surgery; (2) significant liver and renal dysfunction; (3) malignant tumors with a life expectancy of less than 1 year; (4) baseline glucocorticoid drug therapy; and (5) inadequate essential clinical data for METS-IR index calculation. The study received approval from the Ethics Committee of the Third People’s Hospital of Chengdu and adhered strictly to the principles of the Helsinki Declaration. Written informed consent was obtained from all participants enrolled in the study.

### Data collection and definitions

Data were extracted from electronic medical records. The collected variables included sociodemographic information (gender, age, smoking and drinking status), clinical characteristics (type and duration of AF, laboratory results, and details of treatments or procedures), and past medical history, such as hypertension, diabetes mellitus (DM), dyslipidemia, coronary heart disease, HF, stroke, peripheral artery disease, and chronic obstructive pulmonary disease (COPD).

Peripheral venous blood samples were obtained after an overnight fast of at least 8 hours. The following parameters were measured using standard biochemical techniques at the Clinical Laboratory of the Third People’s Hospital of Chengdu: hemoglobin (HGB), fasting blood glucose (FBG), triglycerides (TG), total cholesterol (TC), low-density lipoprotein cholesterol (LDL-C), high-density lipoprotein cholesterol (HDL-C), white blood cell count (WBC) with differentials (neutrophils, lymphocytes, monocytes, eosinophils, basophils), platelet count (PLT), serum creatinine (SCr), and brain natriuretic peptide (BNP). Echocardiography was performed to assess cardiac structure and function, including left atrial diameter (LAD), right atrial diameter (RAD), left ventricular end-diastolic dimension (LVED), and left ventricular ejection fraction (LVEF).

Paroxysmal AF was characterized by AF that spontaneously terminated within 7 days, typically within 48 hours. Persistent AF was defined as atrial fibrillation lasting longer than 7 days (14). Hypertension was diagnosed by the use of antihypertensive medication, or systolic blood pressure ≥140 mmHg, and/or diastolic blood pressure ≥90 mmHg. DM was diagnosed based on hypoglycemic medication use, or casual blood glucose levels ≥11.1 mmol/L, or fasting blood glucose levels ≥7.0 mmol/L, or 2-hour blood glucose levels ≥11.1 mmol/L in the 75-g oral glucose tolerance test (15). BMI was calculated using the formula: weight/height^2^. The IR indexes were determined as the following formula: TyG index = Ln [TG (mg/dl)×FBG (mg/dl) ÷ 2]; METS-IR = Ln [(2×FBG (mg/dl)) + TG (mg/dl)]×BMI (kg/m^2^) ÷ Ln [HDL-C (mg/dl)] (16). TyG-BMI index = TyG×BMI (kg/m²) (15); TG/HDL-C = TG (mg/dl) ÷ HDL-C (mg/dl).

### Ablation protocol and periprocedural management

The RFCA procedure for AF in our institute has been described before (12). In brief, antral circumferential pulmonary vein isolation was performed in all patients with PAF and nPAF. For nPAF patients, additional ablation strategies beyond PVI were implemented at the operator’s discretion, with options of either linear ablation or substrate modification: linear ablation included BOX, mitral isthmus, or tricuspid isthmus ablation, with bidirectional block as the endpoint; substrate modification, targeting fibrotic substrates, involved ablation of low voltage areas to achieve voltage normalization. If AF persisted following these steps, electrical cardioversion was performed to restore sinus rhythm.

### Follow-up

Patients were scheduled for follow-up visits at 3, 6, and 12 months, and annually thereafter. Follow-up was conducted through outpatient consultations, ascertainment of hospital readmissions, and structured telephone interviews. At each follow-up visit, patients were advised to undergo 24-hour Holter monitoring.

The primary endpoint was the composite of MACEs, defined as all-cause death, late AF recurrence, HF events, or stroke occurring after the 3-month blanking period. While AF recurrence represents the primary procedural failure outcome in electrophysiology trials (18), a substantial proportion of post-ablation patients remain at risk of mortality, stroke, and HF hospitalization, which carry independent prognostic significance beyond arrhythmia recurrence alone (19, 20). The composite endpoint was therefore designed to capture the full spectrum of long-term cardiovascular morbidity and mortality in this population. Secondary endpoints included all-cause death, cardiac death, late AF recurrence, HF events, and nonfatal stroke.

All-cause death refers to death attributed to either cardiac or noncardiac causes. If a death could not be attributed to a noncardiac cause, it was classified as cardiac death. Cardiac death was defined as mortality resulting from myocardial infarction, HF, sudden cardiac death, and cardiac procedures. A stroke was characterized as either ischemic or hemorrhagic if detected during the follow-up period, with confirmation through imaging and diagnosis by a neurologist. Late AF recurrence was defined as the identification of any atrial tachyarrhythmia lasting more than 30 seconds by an ECG or Holter monitoring device after the 3-month blank period (17). All events were documented and cross-checked by referencing pertinent medical records whenever possible.

### Statistical analysis

Normality of data distribution was evaluated using the Kolmogorov–Smirnov test. Continuous variables are expressed as mean ± standard deviation (SD) or median with interquartile range (IQR), as appropriate. Group comparisons for continuous variables were conducted using the Student’s t-test or Mann–Whitney U test, while categorical variables, reported as frequencies and percentages, were compared using the chi-square test or Fisher’s exact test, as applicable.

Variables for inclusion in the multivariable model were selected using LASSO-penalized Cox regression, which simultaneously performs variable selection and regularization. The optimal penalty parameter (λ) was determined by 10-fold cross-validation (**Supplementary Figure S2**). This approach identified gender, peripheral arterial disease, basophil count, serum creatinine, and left atrial diameter (LAD) as independent predictors. In addition, four clinically established confounders—age, AF type, hypertension, and diabetes mellitus—were included in the basic model regardless of statistical screening results. All included covariates were assessed for multicollinearity using the Variance Inflation Factor (VIF); all VIF values were below 5 (**Supplementary Figure S3**), confirming the absence of problematic multicollinearity.

The association between the four IR indices and the risk of late MACEs was analyzed using multivariable Cox proportional hazards regression. Three models were constructed to account for confounding: Model 1 was unadjusted. Model 2 was adjusted for a core set of confounders (age, gender, AF type, history of peripheral arterial disease, hypertension, and diabetes mellitus). Model 3 was the fully adjusted model, additionally incorporating basophil count, serum creatinine, and LAD.

Additionally, the dose-response relationships between METS-IR, TG/HDL-C, TyG-BMI, and MACEs were examined using restricted cubic spline (RCS) curves within the Cox regression framework, accompanied by threshold effect analysis. Event-free survival was estimated using the Kaplan-Meier method, with patients categorized by the optimal cutoff values of METS-IR, TG/HDL-C, and TyG-BMI; between-group differences were assessed with the log-rank test.

The optimal cutoff values for METS-IR, TG/HDL-C, and TyG-BMI were determined using the maximally selected log-rank statistics method, implemented via the maxstat package in R. All potential continuous values of each index were evaluated as candidate thresholds. To control for type I error inflation inherent in this data-driven approach, p-values were adjusted using the Lausen & Schumacher (1992) analytical correction method, with candidate proportions restricted to between 0.10 and 0.90 to exclude extreme quantiles. To assess the stability of the identified cutoff values, 1,000 bootstrap resamples were performed. The 95% bootstrap confidence intervals for each optimal cutoff and the proportion of resamples yielding a cutoff within ±10% of the original value (stability rate) are reported.

The predictive performance of a baseline clinical model (comprising age, gender, AF type, history of peripheral arterial disease, TC, basophil count, Scr, and LAD) was evaluated using receiver operating characteristic (ROC) curves. The incremental predictive value of adding BMI or each IR index to this baseline model was quantified by the change in the C-statistic, continuous net reclassification improvement (NRI), and integrated discrimination improvement (IDI). Decision curve analysis (DCA) was conducted to evaluate the clinical utility of each prediction model across a range of threshold probabilities.

Subgroup analyses were performed to assess potential interactions across the following strata: age (<65 vs. ≥65 years), gender, time since AF onset (<1 vs. ≥1 year), AF type (paroxysmal AF [PAF] vs. non-paroxysmal AF), hypertension status, diabetes status, BNP level (<100 vs. ≥100 pg/mL), and left atrial diameter (<40 vs. ≥40 mm).

All statistical analyses were performed using R software version 4.3.2 (https://www.r-project.org/). A two-tailed P value < 0.05 was considered statistically significant.

## Results

### Baseline characteristics

A total of 922 patients with AF who underwent RFCA completed the scheduled follow-up and were included in the final analysis. The cohort had a median age of 67 years (interquartile range [IQR], 59–73), with an equal sex distribution (50% female). During a median follow-up of 36 months (IQR, 16–60), MACEs occurred in 242 patients (26.25%).

Patients who developed MACEs were more likely to be female and presented with a higher prevalence of persistent AF, longer AF duration, elevated BMI, increased BNP, along with LAD and RAD, and higher CHA_2_DS_2_-VASc scores. This group also demonstrated a greater incidence of congestive heart failure (26.90%), stroke (9.90%), and peripheral artery disease (6.60%), but lower levels of TC, HDL-C, and hemoglobin, and reduced use of anticoagulant therapy.

Notably, the levels of non-insulin-based IR indices, such as METS-IR, the TyG index, TG/HDL-C ratio, and TyG-BMI index—were significantly higher among patients with MACEs. A total of 188 AF recurrences, 15 HF readmissions, 31 new-onset HF events, 30 strokes, and 16 deaths from all causes were recorded during the follow-up period.

### Predictive value of IR indices for MACEs risk

Multivariable Cox regression was performed to identify predictors of MACE (**Table 2**). In univariate analysis, age, female sex, non-paroxysmal AF, stroke, peripheral artery disease, heart failure, basophil count, SCr, TC, HDL-C, LDL-C, BNP, LAD, RAD, and CHA_2_DS_2_-VASc score were significantly associated with MACE risk (all P < 0.05). After multivariate adjustment, female sex, peripheral artery disease, basophil count, SCr, and LAD remained independent predictors of MACE (all P < 0.05). Multivariable Cox models (**Table 3**) indicated that METS-IR and the TyG-BMI index were consistently associated with elevated MACEs risk across all models (all P < 0.001). The TG/HDL-C ratio did not reach statistical significance in any model (fully adjusted HR 1.082, 95% CI 0.983–1.192; P = 0.108), nor did the TyG index.

**Table 1:** Baseline characteristics.

Characteristics	Category	Overall	MACEs (-)	MACEs (+)	P value
		(n=922)	(n=680)	(n=242)	
Age, years		67.00[59.00,73.00]	67.00[58.00,73.00]	70.00[63.00,75.00]	0.001
Gender (%)	Male	461(50.00)	361(53.1)	100(41.3)	0.002
	Female	461(50.00)	319(46.9)	142(58.7)	
AF type (%)	PAF	512(55.53)	413(60.7)	99(40.9)	<0.001
	nPAF	410(44.47)	267(39.3)	143(59.1)	
BMI, kg/m2		24.45[22.49,26.57]	24.22[22.27,26.34]	25.12[22.87,27.36]	<0.001
SBP,mmHg		127.00[114.25,139.00]	127.00[115.00,138.00]	127.50[114.00,140.00]	0.513
DBP,mmHg		78.00[71.00,86.08]	78.00[71.00,86.22]	78.00[70.00,86.08]	0.52
Time since the onset, days		468.00[108.00,1728.00]	432.00[108.00,1488.00]	864.00[192.00,2214.00]	0.002
Medical history					
Smoking,n(%)	Never	646(70.07)	467(68.7)	179(74.0)	0.147
	Sometimes	142(15.40)	114(16.8)	28(11.6)	
	Usually	134(14.53)	99(14.6)	35(14.5)	
Drinking,n(%)	Never	671(72.78)	494(72.6)	177(73.1)	0.761
	Sometimes	246(26.68)	183(26.9)	63(26.0)	
	Usually	5(0.54)	3(0.4)	2(0.8)	
Coronary heart disease,n(%)	Yes	105(11.39)	76(11.2)	29(12.0)	0.825
COPD,n(%)	Yes	31(3.36)	23(3.4)	8(3.3)	1
Heart failure,n(%)	Yes	170(18.44)	105(15.4)	65(26.9)	<0.001
Hypertension,n(%)	Yes	501(54.34)	368(54.1)	133(55.0)	0.88
Diabetes mellitus,n(%)	Yes	195(21.15)	136(20.0)	59(24.4)	0.18
Dyslipidemia,n(%)	Yes	174(18.87)	128(18.8)	46(19.0)	1
Stroke,n(%)	Yes	63(6.83)	39(5.7)	24(9.9)	0.039
Peripheral arteries,n(%)	Yes	25(2.71)	9(1.3)	16(6.6)	<0.001
TC,mmol/L		4.17[3.48,4.93]	4.19[3.51,5.03]	4.06[3.41,4.70]	0.035
TG,mmol/L		1.24 [0.93, 1.70]	1.22 [0.91, 1.66]	1.29 [0.96, 1.79]	0.073
HDL-C,mmol/L		1.26[1.06,1.49]	1.28[1.07,1.50]	1.23[1.03,1.44]	0.044
LDL-C,mmol/L		2.38[1.88,2.92]	2.39[1.91,2.95]	2.36[1.77,2.76]	0.073
WBC, ×10^9^/L		5.90[4.92,7.06]	5.84[4.93,7.07]	6.09[4.90,7.01]	0.611
Basophil, ×10^9^/L		0.03[0.02,0.04]	0.03[0.02,0.04]	0.03[0.02,0.04]	0.644
HGB,g/L		137.00[125.00,148.09]	138.00[126.00,150.00]	132.00[121.25,145.00]	<0.001
SCr,μmol/L		73.75[63.90,85.90]	73.35[63.30,85.43]	74.40[65.40,88.10]	0.169
BNP, pg/mL		121.97[59.85,232.20]	107.95[47.98,213.88]	159.65[86.85,300.32]	<0.001
LVEF (%)		60.00[57.00,62.00]	60.00[57.00,62.00]	60.00[57.00,62.00]	0.667
LAD, mm		41.00[37.00,45.00]	40.00[37.00,44.00]	43.00[40.00,46.00]	<0.001
CHA_2_DS_2_-VAScscore		3.00[2.00,4.00]	3.00[1.00,4.00]	3.00[2.00,4.00]	<0.001
METS-IR		42.54[37.75,48.07]	41.91[36.88,47.33]	44.36[39.44,49.88]	<0.001
TyGindex		7.01[6.65,7.34]	6.99[6.63,7.31]	7.06[6.71,7.41]	0.035
TG/HDL-C		0.97[0.69,1.47]	0.95[0.68,1.45]	1.08[0.72,1.58]	0.031
TyG-BMIindex		171.59[152.79,191.33]	169.59[150.75,188.31]	178.05[160.00,199.15]	<0.001

Data were expressed as mean ± SD, median with 25th and 75th percentile or n (%).

AF, atrial fibrillation; nPAF, nonparoxysmal atrial fibrillation; BMI, body mass index; SBP, systolic blood pressure; DBP, diastolic blood pressure; COPD, chronic obstructive pulmonary disease; HF, heart failure; TC, total cholesterol; TG; triglyceride; HDL-C, high-density lipoprotein; LDL-C, low-density lipoprotein cholesterol; WBC, white blood cell count; HGB; hemoglobin; SCr, serum creatinine; BNP, brain natriuretic peptide; LVEF, left ventricular ejection fraction; LAD, left atrial diameter; METS-IR, metabolic score for insulin resistance; TyG, triglyceride-glucose; TG/HDL-C, triglyceride to high-density lipoprotein cholesterol ratio; TyG-BMI, triglyceride glucose-body mass.

**Table 2:** Univariate and multivariate Cox regression analysis for predicting the MACEs recurrence in the overall population.

Variables	Category	Univariate analysis			Multivariate analysis		
		HR	95% CI	P value	HR	95% CI	P value
Age		1.021	1.008–1.035	0.002	1.007	0.985–1.030	0.527
Gender	Male	Ref			Ref		
	Female	1.532	1.186–1.979	0.001	1.588	1.059–2.381	**0.025**
AF type	PAF	Ref			Ref		
	nPAF	1.851	1.432–2.393	<0.001	1.278	0.921–1.773	0.142
Stroke		1.900	1.244–2.903	0.003	1.280	0.780–2.099	0.329
Peripheral arteries		2.922	1.757–4.860	<0.001	2.397	1.330–4.320	**0.004**
HF		1.798	1.352–2.392	<0.001	1.054	0.716–1.552	0.789
Basophil		13.168	2.280–76.053	0.004	12.161	2.033–72.729	**0.006**
HBG		0.989	0.982–0.997	0.004	0.994	0.986–1.002	0.165
SCr		1.009	1.003–1.015	0.003	1.011	1.005–1.017	**0.001**
TC		0.871	0.767–0.989	0.033			
HDL-C		0.647	0.433–0.966	0.033			
LDL-C		0.839	0.705–0.998	0.048			
BNP		1.001	1.000–1.001	<0.001			
LAD		1.069	1.046–1.092	<0.001	1.042	1.013–1.071	**0.005**
RAD		1.046	1.026–1.067	<0.001	1.016	0.990–1.044	0.236
CHA_2_DS_2_-VASc score		1.247	1.148–1.355	<0.001	1.050	0.828–1.333	0.686
Diuretics		1.418	1.050–1.917	0.023			

HR, hazard ratio; CI, confidence interval; RAD, right atrial diameter, other abbreviations can be seen in **Table 1**.

Bold values indicate statistical significance (P < 0.05).

**Table 3:** Association between insulin resistance indices and MACEs in the overall population.

Variables	HR (95% CI)a	P value	HR (95% CI)b	P value	HR (95% CI)c	P value
BMI	1.077(1.040-1.116)	<0.001	1.099(1.058-1.141)	<0.001	1.083(1.042-1.127)	<0.001
METS-IR	1.031(1.016-1.047)	<0.001	1.040(1.023-1.057)	<0.001	1.034(1.016-1.051)	<0.001
TyG index	1.208(0.978-1.493)	0.080	1.148(0.916-1.439)	0.232	1.134(0.901-1.427)	0.285
TG/HDL-C	1.068(0.977-1.167)	0.147	1.079(0.983-1.185)	0.110	1.082(0.983-1.192)	0.108
TyG-BMI index	1.008(1.004-1.012)	<0.001	1.010(1.005-1.014)	<0.001	1.008(1.004-1.013)	<0.001

HR, hazard ratio; CI, confidence interval; other abbreviations can be seen in **Table 1**.

^a^ Model 1: Unadjusted.

^b^ Model 2: Adjusted for age, sex, AF type, history of peripheral arterial disease, hypertension, diabetes mellitus.

^c^ Model 3: Fully adjusted (Model 2 + LA, creatinine, and basophil).

**Figure 1:**
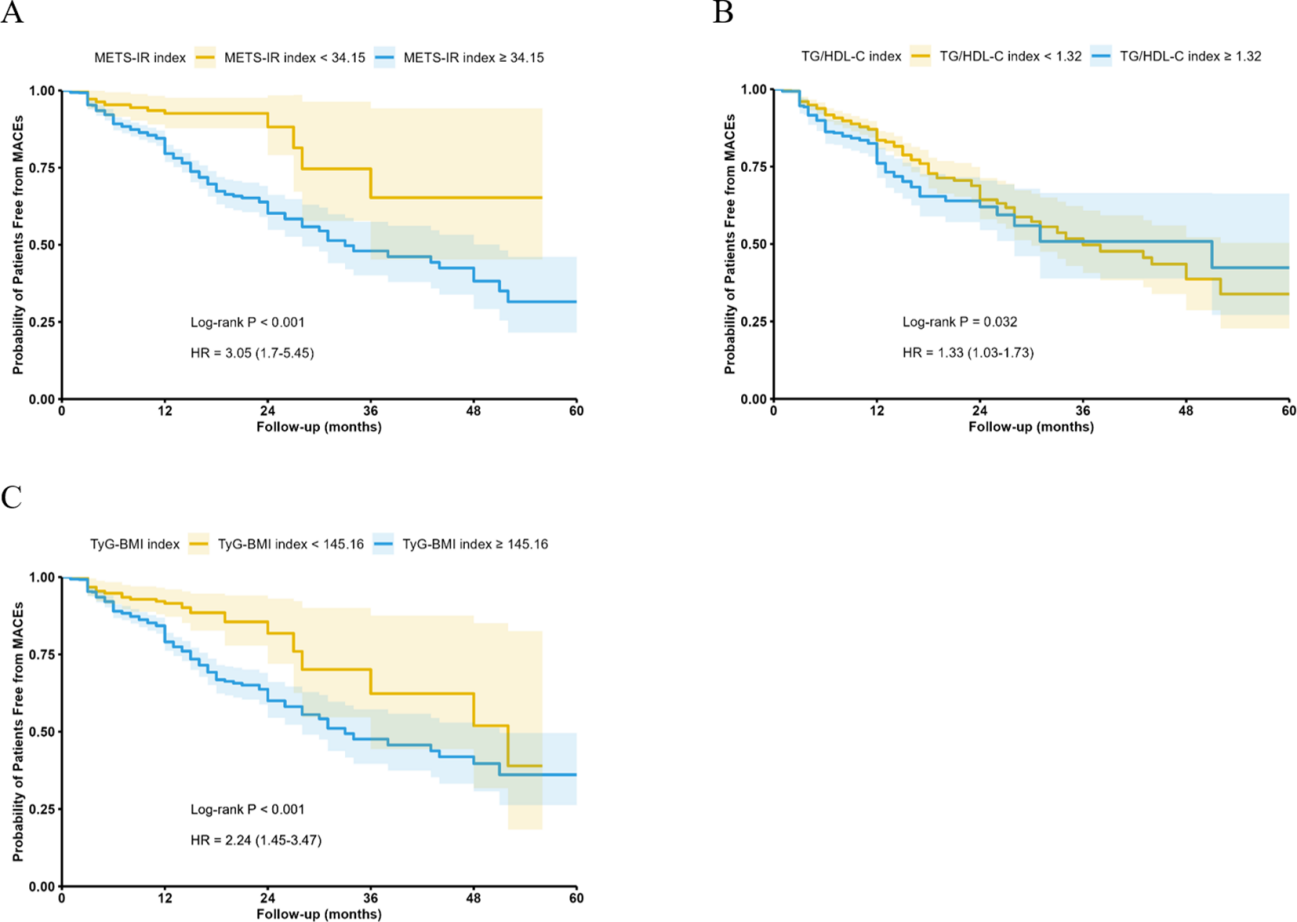
Freedom from MACEs over Time Shown are Kaplan–Meier estimates by the optimal cutoff value of **(A)** METS-IR, **(B)** TG/HDL-C, and **(C)** TyG-BMI index. HR, indicates hazard ratio, METS-IR, metabolic score for insulin resistance; TG/HDL-C, triglyceride to high-density lipoprotein cholesterol ratio; TyG-BMI, triglyceride glucosebody mass index.

Kaplan-Meier analysis, based on the fully adjusted model, illustrated the influence of significant IR indices on event-free survival. Stratification by predefined cut-offs showed that patients with higher index values had significantly reduced event-free survival probabilities (log-rank test, all P < 0.05; **Figure 1**). Separate Kaplan–Meier analyses for each individual endpoint component (all-cause mortality, ischemic stroke, heart failure events, and AF recurrence) are presented in **Supplementary Figure S1**. Component-specific results indicated that the between-quartile differences in METS-IR were predominantly driven by AF recurrence events (log-rank p < 0.001), whereas all-cause mortality (n = 16), stroke (n = 30), and heart failure events (n = 46) showed no significant between-group differences (all log-rank p > 0.05). In a pre-specified sensitivity analysis restricted to hard endpoints only (all-cause mortality, ischemic stroke, and heart failure rehospitalization; n = 73 events; **Supplementary Tables S1**–**S3**), METS-IR did not reach statistical significance in the fully adjusted model (adjusted HR = 0.982, 95% CI: 0.950–1.014, P = 0.256).

### Assessment of nonlinear relationships

**Figure 2:**
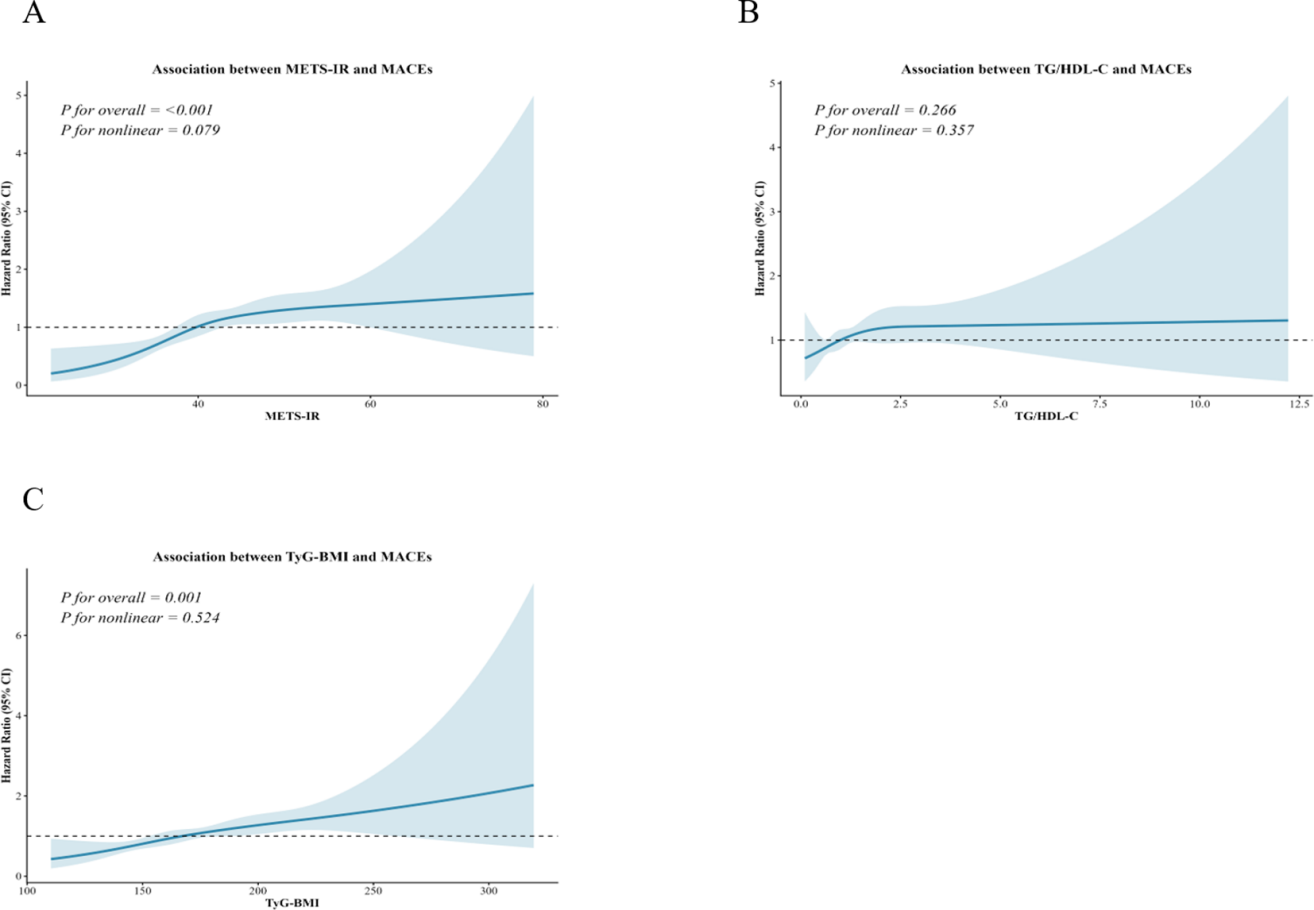
Restricted cubic spline curves for MACE by **(A)** METS-IR, **(B)** TG/HDL-C, and **(C)** TyG-BMI index. *HR* are indicated by solid lines and 95% CIs by shaded areas. CI, confidence interval; METS-IR, metabolic score for insulin resistance; TG/HDL-C, triglyceride to high-density lipoprotein cholesterol ratio; TyG-BMI, triglyceride glucosebody mass index.

RCS analyses were applied to examine nonlinear associations between three IR indices—METS-IR, TyG-BMI index, and TG/HDL-C ratio—and MACEs risk (**Figure 2**). METS-IR demonstrated an approximately linear dose-response relationship with MACEs (P for overall < 0.001, P for nonlinear = 0.079; **Figure 2**). The TG/HDL-C ratio demonstrated no significant overall association with MACEs risk (P for overall = 0.266, P for nonlinear = 0.357; **Figure 2**). The TyG-BMI index also showed a significant linear association with MACEs (P for overall < 0.001, P for nonlinear = 0.524; **Figure 2**).

### Incremental predictive value of BMI and IR-related indices

**Figure 3:**
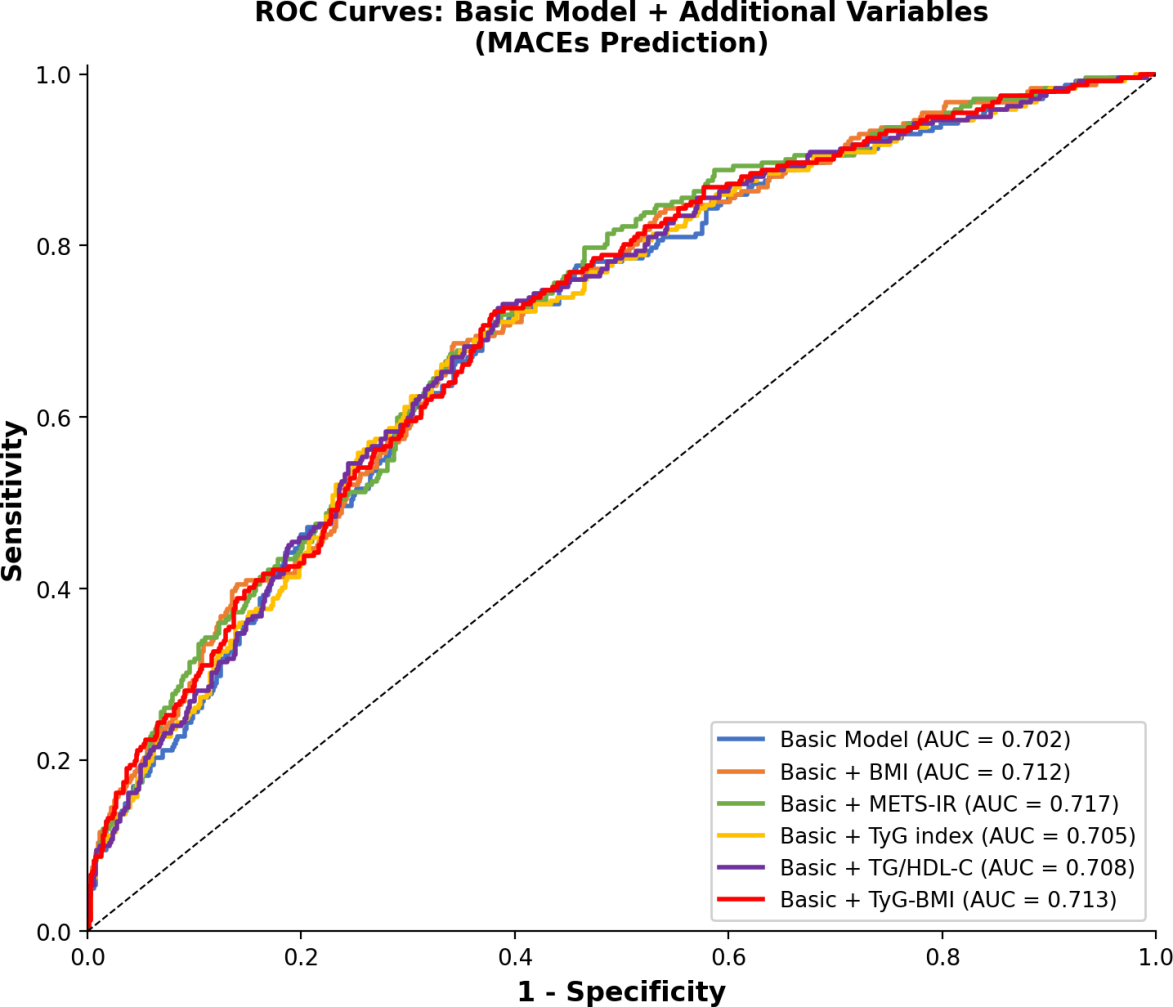
The receiver operating characteristic curves of the BMI and IR indices as a marker to predict late MACEs. Basic Model (AUC = 0.702); Basic + BMI (AUC = 0.712, P = 0.016); Basic + METS-IR (AUC = 0.717, P<0.001); Basic + TyG (AUC = 0.705, P = 0.200); Basic + TG/HDL-C (AUC = 0.708, P = 0.092); Basic + TyG-BMI (AUC = 0.713, P=<0.001).

We evaluated the incremental prognostic value of BMI and four IR indices (METS-IR, TyG index, TG/HDL-C, TyG-BMI) by adding each separately to a basic clinical model including age, gender, AF type, hypertension, diabetes mellitus, peripheral arterial disease, basophil count, SCr, and LAD. The C-statistic of the basic model was 0.702 (95% CI: 0.673–0.746). Incorporation of BMI, METS-IR, TG/HDL-C, and TyG-BMI significantly improved predictive discrimination, with C-statistics increased to 0.712, 0.717, 0.708, and 0.713, respectively (all P < 0.05). The TyG index yielded no significant improvement in C-statistic (P = 0.200) (**Table 4**, **Figure 3**).

**Table 4:** Evaluation of the incremental prognostic value of adding IR indices to the basic model to predict clinical outcomes.

Model	C-statistic (95% CI)	P value	NRI (95% CI)	P value	IDI (95% CI)	P value
Basic model	0.702 (0.673-0.746)	Ref.	Ref.		Ref.	
+ BMI	0.712 (0.684-0.755)	0.016	0.187 (0.055-0.360)	0.004	0.012 (0.003-0.033)	<0.001
+ METS-IR	0.717 (0.689-0.757)	<0.001	0.291 (0.126-0.426)	<0.001	0.015 (0.004-0.035)	0.004
+ TyG index	0.705 (0.677-0.750)	0.200	0.052 (-0.077-0.211)	0.432	0.003 (0.000-0.014)	0.052
+ TG/HDL-C	0.708 (0.681-0.752)	0.092	0.200 (0.041-0.344)	0.024	0.006 (0.000-0.021)	<0.001
+ TyG-BMI index	0.713 (0.685-0.757)	<0.001	0.205 (0.047-0.359)	0.012	0.013 (0.002-0.031)	0.004

The basic model included age, gender, AF type, hypertension, diabetes mellitus, peripheral arterial diseases, basophil, SCr, and LAD. CI, confidence interval; NRI, net reclassification improvement; ID, I integrated discrimination improvement; other abbreviations can be seen in **Table 1**.

NRI and IDI analyses further confirmed the incremental value of these markers (**Table 4**). METS-IR conferred the greatest predictive gain, significantly elevating the C-statistic, NRI (0.291, 95% CI: 0.126–0.426, P < 0.001), and IDI (0.015, 95% CI: 0.004–0.035, P = 0.004). TyG-BMI significantly improved NRI (0.205, 95% CI: 0.047–0.359, P = 0.012) and IDI (0.013, 95% CI: 0.002–0.031, P = 0.004). BMI also produced significant increases in NRI (0.187, P = 0.004) and IDI (0.012, P < 0.001). TG/HDL-C showed significant improvements in NRI and IDI despite a non-significant change in C-statistic (P = 0.092). The TyG index failed to improve any discrimination or reclassification indices significantly.

**Figure 4:**
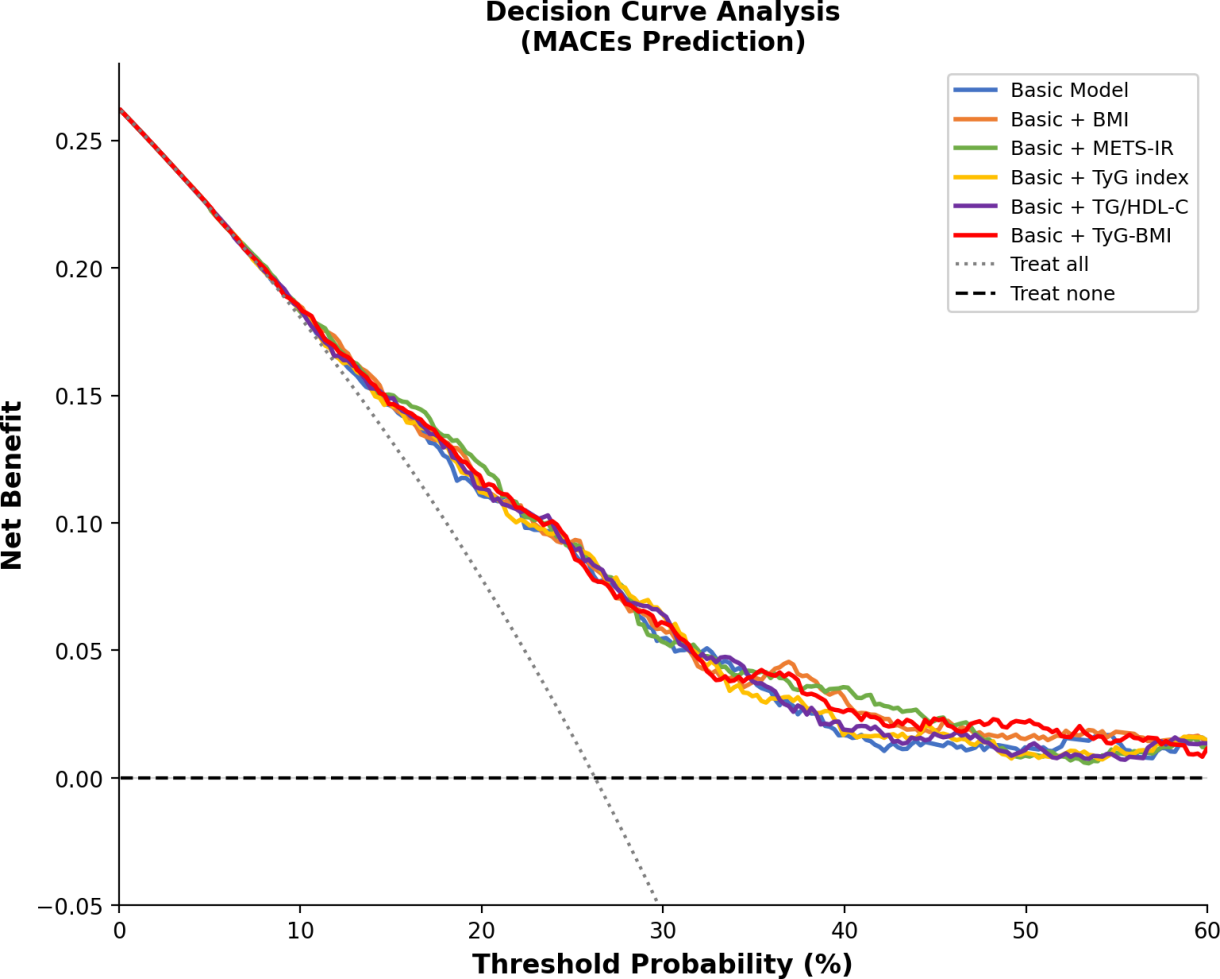
Decision curve analysis demonstrating the net benefit of the Basic Model and each IR index–augmented model for predicting MACEs.

DCA showed that all extended models yielded positive net clinical benefit across threshold probabilities of approximately 5%–45%, outperforming the “treat-all” and “treat-none” strategies (**Figure 4**). The models incorporating METS-IR and TyG-BMI exhibited the highest net benefit throughout most clinical probability ranges.

### Subgroup analyses of METS-IR for MACEs risk

**Figure 5:**
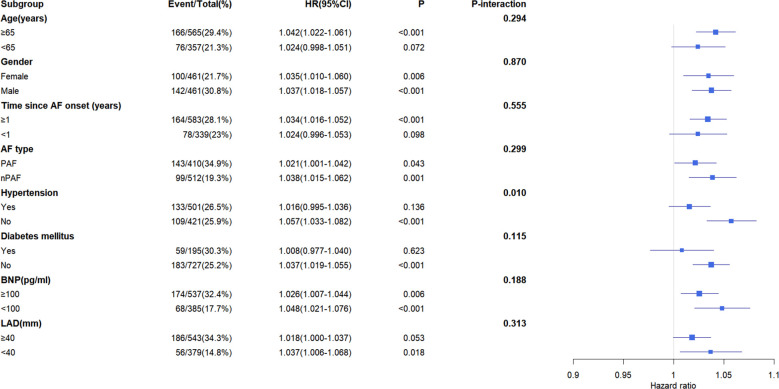
Subgroups analyses of METS-IR for MACEs. Hazard ratios are presented as per 1.0-SD increase in the METS-IR for MACEs. CI, confidence interval; METS-IR, metabolic score for insulin resistance; BMI, body mass index; AF, atrial fibrillation; PAF, paroxysmal atrial fibrillation; nPAF, nonparoxysmal atrial fibrillation; BNP, brain natriuretic peptide; LAD, left atrial diameter.

Subgroup analyses were performed to further examine the METS-IR–MACEs association and identify potential effect modifiers (**Figure 5**). A significant positive association was observed in most subgroups, including older patients (≥65 years; HR 1.042, 95% CI 1.022–1.061), both sexes, patients with longer AF duration (≥1 year; HR 1.034, 95% CI 1.016–1.052), and those with persistent AF (HR 1.038, 95% CI 1.015–1.062). No significant association was detected in younger patients, those with shorter AF duration, or patients with comorbid hypertension or diabetes.

A significant interaction was identified for hypertension (P for interaction = 0.010). The hazard ratio for MACEs per unit increase in METS-IR was significantly elevated in non-hypertensive patients (HR 1.057, 95% CI 1.033–1.082, P < 0.001) compared to hypertensive patients (HR 1.016, 95% CI 0.995–1.038, P = 0.138). No significant interactions were observed for other subgroups—including age, sex, AF duration, AF type, diabetes, BNP level, or LAD (all P for interaction > 0.05)—supporting the consistency of the primary outcome.

## Discussion

Given the limited evidence regarding the predictive value of IR indices in AF patients following RFCA, the results of the present study demonstrated that elevated levels of IR, indicated by METS-IR and TyG-BMI index, were significantly associated with increased MACEs risk post-ablation. This study is the first to directly compare the prognostic value of four IR indices for long-term MACEs after RFCA. The key finding is that METS-IR outperforms the others, providing a clinically significant improvement in the predictive performance of standard risk models.

Catheter ablation is a well-established treatment for AF, with evidence confirming its efficacy in reducing mortality, HF hospitalization rates, and improving patients’ quality of life (18). However, a substantial proportion of patients remain at residual risk of adverse outcomes following the procedure. Clinical trial data highlight this concern: one study reported composite endpoint rates (AF recurrence and stroke) of approximately 50% (19), while another study documented HF rehospitalization and all-cause mortality rates of 20.7% and 13.4%, respectively (20). These MACEs trigger a detrimental cascade, resulting in reduced quality of life, increased healthcare utilization, heightened dependence on antiarrhythmic drugs and repeat procedures, and elevated long-term mortality risk (18, 21, 22). Thus, the early identification of patients at high risk of long-term MACEs is clinically critical. Our previous research identified an association between IR and AF recurrence after catheter ablation (23). Building on this work, the present study demonstrates that IR also exhibits significant predictive value for broader MACEs following AF ablation.

The assessment of IR is critical for optimizing risk stratification and guiding targeted preventive strategies (24). Multiple validated surrogate indices have been developed, including the TyG index, METS-IR, TyG-BMI, and TG/HDL-C ratio (16). Among these indices, METS-IR has demonstrated superior predictive performance for AF recurrence compared with other surrogate markers (11). Consistent with this evidence, our findings confirm that prediction models incorporating METS-IR yield enhanced accuracy in evaluating the risk of longterm MACEs following AF ablation.

The predictive capacity of METS-IR originates from its integrated assessment of four key metabolic parameters: BMI, FBG, TG, and HDL-C. This comprehensive design effectively captures the pathophysiological crosstalk between lipid and glucose metabolism, which underlies arrhythmogenesis and cardiovascular complications. Specifically, elevated BMI is associated with increased AF recurrence after catheter ablation (25). Lipid abnormalities further augment this risk: meta-analyses have illustrated an inverse association between HDL-C levels and AF incidence (26), while elevated TG levels predict both AF recurrence and stroke (27, 28).

The TyG index did not reach statistical significance as an independent predictor of MACE in this cohort (fully adjusted HR = 1.134, 95% CI: 0.901–1.427, P = 0.285). This finding contrasts with its established predictive utility in coronary artery disease populations, and several factors may explain the discrepancy. The TyG index captures a narrower metabolic phenotype than METS-IR or TyG-BMI, being derived solely from fasting triglycerides and fasting glucose without incorporating adiposity measures. These observations highlight the importance of incorporating adiposity-related parameters when evaluating IR-mediated cardiovascular risk in AF patients. Furthermore, our analysis relied solely on one-off baseline TyG testing and was limited to a single-center cohort of moderate sample size. Future large-scale multicenter cohorts would help clarify whether longitudinal changes in TyG over follow-up improve its prognostic performance for adverse cardiovascular events.

Critically, IR represents a modifiable therapeutic target. Improving IR has the potential to attenuate underlying atrial remodeling, thereby reducing the risks of AF recurrence, HF, and stroke. This principle is strongly supported by evidence from cardiometabolic therapies. Sodium-glucose cotransporter-2 (SGLT2) inhibitors, for instance, exert robust cardioprotective effects. Large-scale trials and meta-analyses demonstrate that these agents significantly lower the composite risk of HF progression and cardiovascular death (29), with benefits extending beyond glycemic control (30). The EMPEROR-Preserved trial specifically established empagliflozin’s efficacy in reducing cardiovascular death or HF hospitalization in patients with HF and preserved ejection fraction (31). In addition, a recent systematic review and meta-analysis reported that both empagliflozin and dapagliflozin were associated with significant reductions in sudden cardiac death risk in patients with established cardiovascular disease (32). Notably, in patients undergoing AF ablation, SGLT2 inhibitor therapy significantly decreases arrhythmia recurrence and the subsequent need for interventions such as cardioversion or repeat ablation (33). This anti-arrhythmic effect is also observed in glucagon-like peptide-1 (GLP-1) receptor agonists. Treatment with semaglutide following catheter ablation is associated with a sustained reduction in atrial arrhythmia recurrence over 12 months (34). Collectively, these findings validate that pharmacologically targeting IR can directly improve clinical outcomes in patients after AF ablation.

The present cohort comprised exclusively radiofrequency catheter ablation procedures. Emerging evidence suggests that ablation modality—including cryoablation and pulsed field ablation—may independently influence arrhythmia recurrence rates and patient-reported quality of life outcomes (35). Whether the association between IR status and MACE observed in the present study is modified by ablation energy source therefore remains to be established. Future research involving multi-modality cohorts should formally examine this interaction, particularly as pulsed field ablation gains wider clinical adoption. IR indices in AF management have also demonstrated broader utility: a recent study found that elevated triglyceride-glucose–related parameters independently predict failed cardioversion in AF patients (36), supporting their value across the spectrum of AF treatment strategies.

## Limitations

This study has several limitations inherent to its design. First, as a retrospective single-center analysis, it cannot establish a causal relationship between IR and MACEs, and residual confounding from unmeasured variables cannot be ruled out. Second, AF recurrence was assessed via intermittent monitoring instead of continuous rhythm surveillance, which likely underestimated asymptomatic recurrences and may thus have weakened the observed associations. Third, the generalizability of the findings is constrained by the exclusive recruitment of an ethnic Chinese cohort and the lack of external validation. Therefore, the proposed risk thresholds need to be prospectively validated in large, diverse, multiethnic populations. Fourth, ablation strategy (pulmonary vein isolation alone versus additional linear or substrate ablation) was not formally adjusted for in the primary model, given its close collinearity with AF type in this cohort; operator-specific factors such as lesion quality and contact force were also not captured and may represent residual sources of heterogeneity. Of note, further stratified analyses within the nPAF subset to dissect whether different ablation strategies modify the IR–MACE relationship would be clinically valuable, and we acknowledge this as an important direction for future dedicated investigations. Fifth, the cohort comprised exclusively radiofrequency catheter ablation procedures; the potential interaction between IR status and ablation modality on MACE outcomes remains to be investigated in future multi-energy-source studies. Sixth, hard cardiovascular endpoints (mortality, stroke, and heart failure event) were not independently predicted by METS-IR in this sample, likely owing to insufficient event numbers.

## Conclusion

Among the evaluated non-insulin-based IR indices, METS-IR demonstrated a statistically significant, albeit modest, incremental improvement in the prediction of composite MACE in AF patients following RFCA (C-statistic: 0.702 to 0.717, p < 0.001). This finding supports METS-IR as a low-cost, readily available supplementary risk stratification tool; however, the magnitude of this discrimination improvement does not justify replacement of established clinical risk models, and prospective validation in independent, ethnically diverse cohorts is required before routine clinical translation. Collectively, these findings support the incorporation of IR assessment into post-ablation clinical care to identify patients who may benefit from metabolic optimization therapy.

## Data Availability

The raw data supporting the conclusions of this article will be made available by the authors, without undue reservation.

## Supplementary material

Supplementary Figure 1 Kaplan–Meier curves for each individual endpoint component—**(A)** stroke, **(B)** all-cause mortality, **(C)** heart failure events, and **(D)** AF recurrence—stratified by METS-IR quartile.

Supplementary Figure 2 LASSO-penalized Cox regression with 10-fold cross-validation. SD, standard deviation; SE, standard error.

Supplementary Figure 3 VIF values for all covariates in each of the six Cox models: the basic model and the basic model with BMI, METS-IR, TyG index, TG/HDL-C, or TyG-BMI index added separately. VIF, variance inflation factor.
