# Supplementary figures and images for: Insulin resistance as a predictor of long-term adverse cardiovascular event risk in patients with atrial fibrillation following radiofrequency catheter ablation
Source: Front Endocrinol (Lausanne). 2026 Jul 2;17:1831673. doi: 10.3389/fendo.2026.1831673 (PMC13372583; doi:10.3389/fendo.2026.1831673)

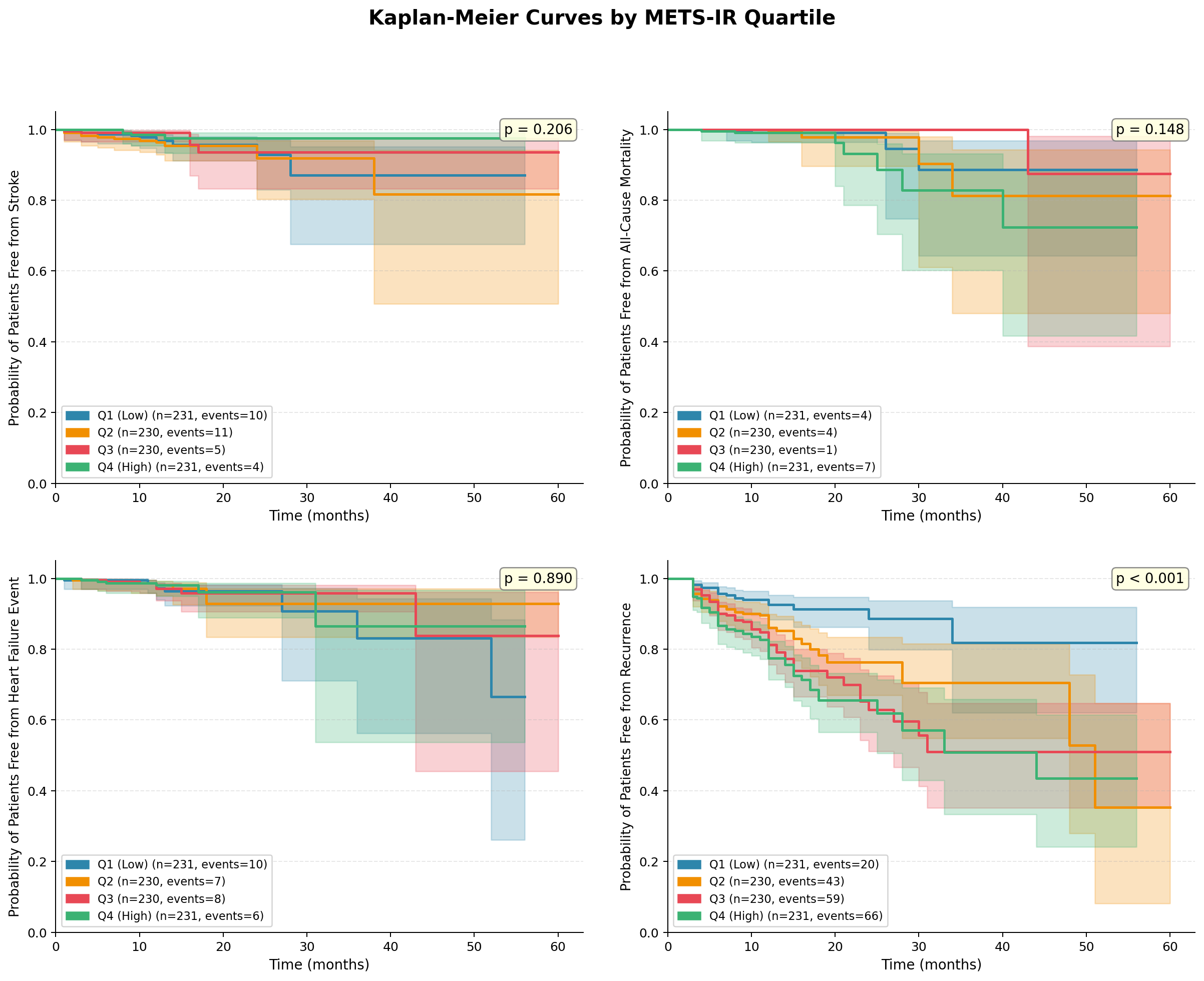

Supplement: Supplementary Figure 1 — Kaplan–Meier curves for each individual endpoint component—(A) stroke, (B) all-cause mortality, (C) heart failure events, and (D) AF recurrence—stratified by METS-IR quartile. [file Image1.tif]

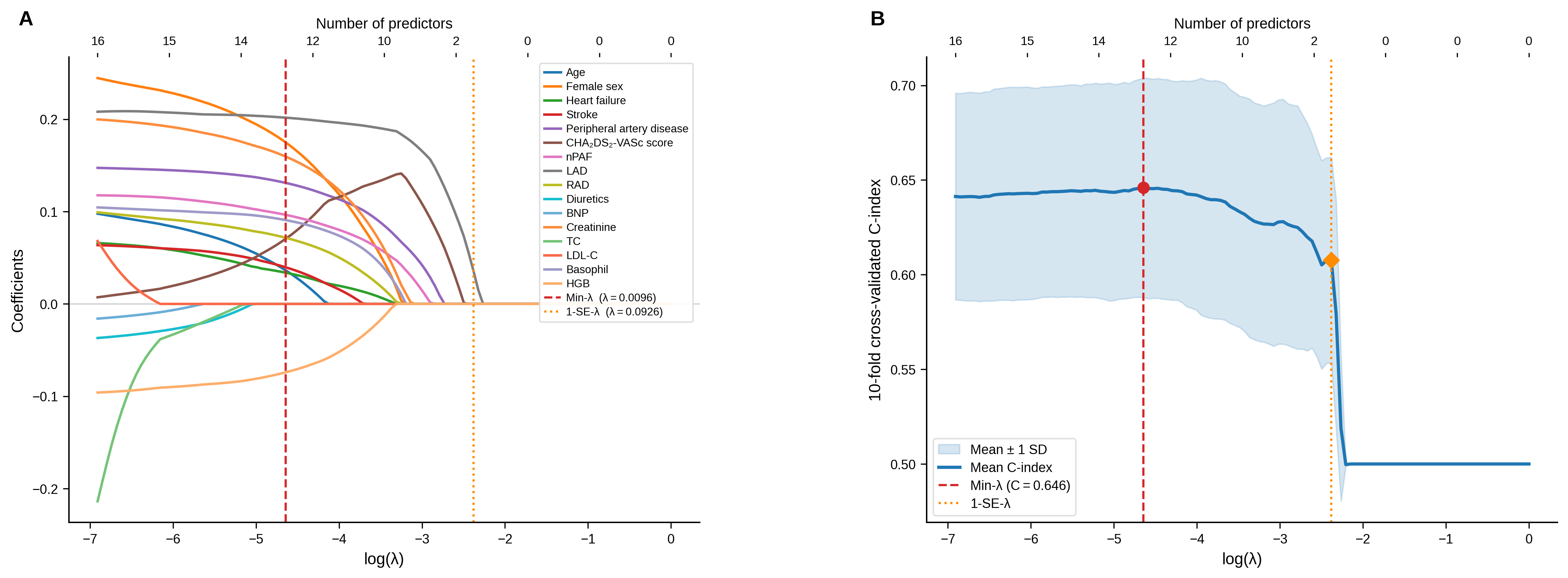

Supplement: Supplementary Figure 2 — LASSO-penalized Cox regression with 10-fold cross-validation. SD, standard deviation; SE, standard error. [file Image2.tiff]

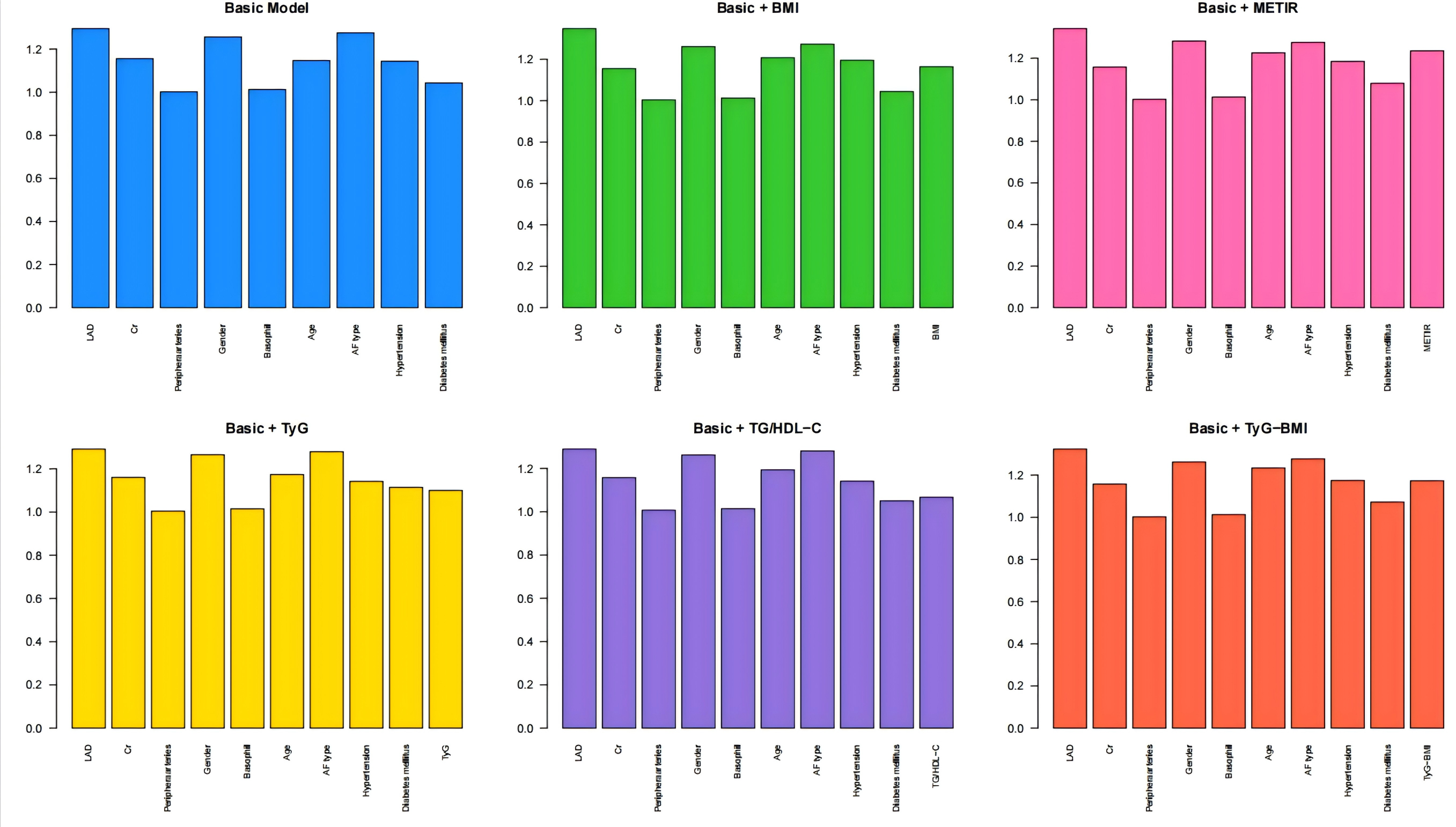

Supplement: Supplementary Figure 3 — VIF values for all covariates in each of the six Cox models: the basic model and the basic model with BMI, METS-IR, TyG index, TG/HDL-C, or TyG-BMI index added separately. VIF, variance inflation factor. [file Image3.tiff]
