# Supplementary material for: Insulin resistance as a predictor of long-term adverse cardiovascular event risk in patients with atrial fibrillation following radiofrequency catheter ablation
Source: Front Endocrinol (Lausanne). 2026 Jul 2;17:1831673. doi: 10.3389/fendo.2026.1831673 (PMC13372583; doi:10.3389/fendo.2026.1831673)
Supplement: Supplementary file 4 [file Table1.docx]

**Supplementary Table1**

| Characteristics |  | Overall | Non-AF MACEs (-) | Non-AF MACEs(+) | Pvalue |
| --- | --- | --- | --- | --- | --- |
|  |  | (n=922) | (n=849) | (n=73) |  |
| Age, years |  | 67.00 [59.00, 73.00] | 67.00 [59.00, 73.00] | 72.00 [65.00, 78.00] | <0.001 |
| Gender (%) | Male | 461 (50.0) | 431 (50.8) | 30 (41.1) | 0.143 |
|  | Female | 461 (50.0) | 418 (49.2) | 43 (58.9) |  |
| AF type(%) | PAF | 512 (55.5) | 482 (56.8) | 30 (41.1) | 0.014 |
|  | nPAF | 410 (44.5) | 367 (43.2) | 43 (58.9) |  |
| BMI, kg/m2 |  | 24.45 [22.49, 26.57] | 24.46 [22.52, 26.58] | 23.51 [22.04, 26.43] | 0.078 |
| SBP,mmHg |  | 127.00 [114.25, 139.00] | 127.00 [115.00, 139.00] | 128.00 [112.00, 138.00] | 0.676 |
| DBP,mmHg |  | 78.00 [71.00, 86.08] | 78.00 [71.00, 87.00] | 78.00 [72.00, 85.00] | 0.862 |
| Time since the onset, days |  | 468.00 [108.00, 1728.00] | 432.00 [108.00, 1728.00] | 864.00 [108.00, 1860.00] | 0.325 |
| Smoking,n(%) | Never | 646 (70.1) | 592 (69.7) | 54 (74.0) | 0.348 |
|  | Sometimes | 142 (15.4) | 135 (15.9) | 7 (9.6) |  |
|  | Usually | 134 (14.5) | 122 (14.4) | 12 (16.4) |  |
| Drinking,n(%) | Never | 671 (72.8) | 617 (72.7) | 54 (74.0) | 0.025 |
|  | Sometimes | 246 (26.7) | 229 (27.0) | 17 (23.3) |  |
|  | Usually | 5 (0.5) | 3 (0.4) | 2 (2.7) |  |
| Coronary heart disease,n(%) | Yes | 105 (11.4) | 95 (11.2) | 10 (13.7) | 0.649 |
| COPD,n(%) | Yes | 31 (3.4) | 29 (3.4) | 2 (2.7) | 1 |
| Heart failure,n(%) | Yes | 170 (18.4) | 140 (16.5) | 30 (41.1) | <0.001 |
| Hypertension,n(%) | Yes | 501 (54.3) | 460 (54.2) | 41 (56.2) | 0.838 |
| Diabetes mellitus,n(%) | Yes | 195 (21.1) | 179 (21.1) | 16 (21.9) | 0.986 |
| Dyslipidemia,n(%) | Yes | 174 (18.9) | 162 (19.1) | 12 (16.4) | 0.691 |
| Stroke,n(%) | Yes | 63 (6.8) | 52 (6.1) | 11 (15.1) | 0.008 |
| Peripheral arteries,n(%) | Yes | 25 (2.7) | 22 (2.6) | 3 (4.1) | 0.696 |
| Laboratory data |  |  |  |  |  |
| TC,mmol/L |  | 4.17 [3.48, 4.93] | 4.18 [3.50, 4.98] | 3.96 [3.30, 4.47] | 0.062 |
| TG,mmol/L |  | 1.24 [0.93, 1.70] | 1.25 [0.93, 1.70] | 1.20 [0.85, 1.67] | 0.422 |
| HDL-C,mmol/L |  | 1.26 [1.06, 1.49] | 1.27 [1.06, 1.49] | 1.25 [1.06, 1.47] | 0.726 |
| LDL-C,mmol/L |  | 2.38 [1.88, 2.92] | 2.38 [1.91, 2.94] | 2.24 [1.71, 2.66] | 0.047 |
| WBC,×10^9^/L |  | 5.90 [4.92, 7.06] | 5.88 [4.91, 7.03] | 6.23 [5.06, 7.20] | 0.218 |
| Basophil,×10^9^/L |  | 0.03 [0.02, 0.04] | 0.03 [0.02, 0.04] | 0.03 [0.02, 0.04] | 0.373 |
| HGB,g/L |  | 137.00 [125.00, 148.09] | 137.00 [125.00, 149.00] | 132.00 [124.00, 144.00] | 0.157 |
| SCr,μmol/L |  | 73.75 [63.90, 85.90] | 73.60 [63.60, 85.70] | 76.80 [68.10, 88.20] | 0.088 |
| BNP, pg/mL |  | 121.97 [59.85, 232.20] | 115.53 [55.40, 223.71] | 173.60 [117.80, 360.70] | <0.001 |
| LVEF(%) |  | 60.00 [57.00, 62.00] | 60.00 [57.00, 62.00] | 58.08 [57.00, 62.00] | 0.043 |
| LAD, mm |  | 41.00 [37.00, 45.00] | 41.00 [37.00, 45.00] | 43.00 [39.00, 47.00] | 0.005 |
| CHA_2_DS_2_-VAScscore |  | 3.00 [2.00, 4.00] | 3.00 [1.00, 4.00] | 4.00 [2.00, 5.00] | <0.001 |
| METS-IR |  | 42.54 [37.75, 48.07] | 42.63 [37.87, 48.25] | 40.44 [36.43, 47.21] | 0.155 |
| TyGindex |  | 7.01 [6.65, 7.34] | 7.01 [6.65, 7.34] | 6.93 [6.63, 7.34] | 0.647 |
| TG/HDL-C |  | 0.97 [0.69, 1.47] | 0.97 [0.70, 1.47] | 0.94 [0.58, 1.53] | 0.44 |
| TyG-BMI index |  | 171.59 [152.79, 191.33] | 172.27 [153.30, 191.55] | 164.57 [147.78, 188.07] | 0.076 |

Abbreviations can be seen in Table 1

**Supplementary Table2**

| Variables | Univariate analysis | | | Multivariate analysis | | |
| --- | --- | --- | --- | --- | --- | --- |
|  | HR | 95% CI | P value | HR | 95% CI | P value |
| Age | 1.047 | 1.019-1.074 | <0.001 | 1.046 | 1.009-1.085 | 0.013 |
| Stroke | 3.294 | 1.721-6.304 | <0.001 | 2.317 | 1.139-4.716 | 0.02 |
| Heart failure | 3.476 | 2.176-5.554 | <0.001 | 2.369 | 1.326-4.232 | 0.004 |
| BNP | 1.001 | 1.000-1.001 | <0.001 | 1 | 0.999-1.001 | 0.833 |
| LVEF | 0.957 | 0.931-0.983 | 0.002 | 0.978 | 0.945-1.013 | 0.217 |
| LAD | 1.069 | 1.028-1.112 | <0.001 | 1.035 | 0.990-1.083 | 0.132 |
| CHA_2_DS_2_-VASc score | 1.385 | 1.187-1.616 | <0.001 | 0.999 | 0.790-1.263 | 0.993 |

*HR hazard ratio, CI confidence interval; abbreviations can be seen in Table 1*

**Supplementary Table3 Association between insulin resistance indices and Non-AF MACEs**

|  | HR (95% CI)a | P value | HR (95% CI)b | P value | HR (95% CI)c | P value |
| --- | --- | --- | --- | --- | --- | --- |
| BMI | 0.953(0.887-1.023) | 0.186 | 0.974(0.906-1.046) | 0.463 | 0.956(0.886-1.033) | 0.254 |
| METS-IR | 0.985(0.956-1.014) | 0.306 | 0.990(0.961-1.020) | 0.514 | 0.982(0.950-1.014) | 0.256 |
| TyG index | 0.877(0.579-1.329) | 0.536 | 0.907(0.598-1.375) | 0.644 | 0.923(0.598-1.426) | 0.720 |
| TG/HDL-C | 0.956(0.773-1.181) | 0.675 | 1.008(0.828-1.228) | 0.933 | 1.018(0.833-1.244) | 0.862 |
| TyG-BMI index | 0.995(0.987-1.003) | 0.181 | 0.997(0.989-1.005) | 0.426 | 0.995(0.987-1.004) | 0.277 |

*HR* hazard ratio, *CI* confidence interval; abbreviations can be seen in Table 1

a Model 1: unadjusted.

b Model 2: adjusted for age, history of heart failure, and history of stroke.

c Model 3: model 2+further adjusted for BNP, EF, left atrial diameter, and CHA_2_DS_2_-VASc score.

**Supplementary Table4 Bootstrap stability of the optimal cutoffs**

| Index | Optimal Cutoff | 95% Bootstrap CI | Stability (within ±10%) |
| --- | --- | --- | --- |
| METS‑IR | 34.15 | 33.45–52.74 | 52.9% |
| TyG‑BMI | 145.16 | 142.02–206.96 | 44.5% |
| TG/HDL‑C | 1.32 | 0.49–1.99 | 19.4% |

*HR* hazard ratio, *CI* confidence interval; abbreviations can be seen in Table 1
